# Experience of treating high risk prostate hyperplasia patients with a HPS120 laser

**DOI:** 10.1186/1471-2490-13-64

**Published:** 2013-11-29

**Authors:** Li-Jun Chen, Hai-Xing Mai, Li Zhao, Nan Qu, Ya-Lin Wang, Chen Huang, Xue-Chao Li, Jin-Kai Dong, Fei Tang, Biao Chen

**Affiliations:** 1 Deptment of Urology, 307 Hospital of PLA, Beijing 100071, China; 2 Department of Urology, Chinese People’s Liberation Army General Hospital & Medical School of Chinese People’s Liberation Army, Beijing 100853, China

**Keywords:** 120 watt green laser, Benign prostatic hyperplasia, Elderly patients

## Abstract

**Background:**

The aim of this study was to investigate the effects and safety of 120 watt PVP surgery for the high risk prostate hyperplasia patients.

**Methods:**

120 watt PVP surgery was performed on 120 cases of high risk prostate hyperplasia patients. The assessment included the operation time, energy consumed, hemoglobin changes, and serum salt concentration, whether to keep urinary catheter, hospitalization time, and complications after the operation. International Prostate Symptom Scoring (IPSS), the maximum urine flow rate (Qmax) and residual urine volume (RUV) were conducted preoperatively and postoperatively for the patients.

**Results:**

There were 30% of patients taking oral anti-coagulant drug (n = 36), 88 cases with abnormal ECGs. All the patient’s internal diseases, include the cardiovascular disease (42/120), the hypertension (56/120), the respiratory system diseases (51/120), the cerebrovascular diseases (39/120), anemia (24/120), liver or kidney dysfunction (16/120), diabetes (18/120), hypoproteinemia (15/120) were under controlled. The average age, prostate volume and energy consumed was 82.8 ± 8.6 (70–96) years, 66.1 ± 25.3 (30–160) ml, and 224 ± 85 (31–596) kJ respectively. The average follow-up time was 20.8 ± 3.2 (18–24) months. The incidence of bladder neck contracture and urethral stricture were 1.7% and 0.8% respectively, no prostate cancer occurred during the subsequent follow-up period.

**Conclusions:**

120 watt PVP surgery can safely and effectively alleviate the urination parameters of high risk prostate hyperplasia patients. The surgical process is safe and effective, and is not affected by the various internal diseases or the use of oral anti-coagulant drugs.

## Background

Although transurethral resection of the prostate (TURP) and various kinds of minimally invasive surgery had being widely used for benign prostatic hyperplasia (BPH) for many years, these treatment methods still have serious complications on patients and sometimes cases of death still occurred postoperatively [1,2]. Because most of these patients are old, and accompanied with many medical complications, Gesenberg [3] believe that approximately 10%-15% of BPH patients cannot tolerate surgical operations owing to concomitant illnesses. These patients could only undergo non-surgical drug treatment to maintain urination or bladder fistulization for urinary diversion, and this seriously affected patients’ quality of life [4,5]. These elderly high risk BPH patients have always constituted a difficult problem in terms of clinical treatment [6]. So, finding a mini-invasive way to reduce the operation time and bleeding during the surgery become the most important direction of our surgeon.

Within the past 10 years, green laser system has already developed from coagulation to incisions and vaporization. This surgical method can not only rapidly vaporize the prostate tissue, but also reduce surgical complication rate to an acceptable level [7-9].

In this study we carried out the 120 watt PVP surgery for the above-described special patients, and the longest follow up time was 24 months. The aim of this article is to discuss how complications associated with photoselective vaporization of the prostate (PVP) compare with these elderly high risk patients. In addition, recommendations and discussion regarding minimizing complications through modification of the new GreenLight HPS (TM) 120-W (American Medical System Incorporation, Minnetonka, MN, USA) laser operative technique are provided.

## Methods

### Patient

Between January 2009 and January 2011, a total of 120 patients with LUTS due to BPH were assessed for eligibility to enter the study. All patients were subjected to the standard urologic preoperative evaluation, including history taking, clinical examination including digital rectal examination (DRE), urine analysis, routine blood chemistry including prostate-specific antigen (PSA). TRUS was used to estimate the size of the prostate. Urine flowery was carried out to measure Qmax. IPSS was completed by self assessment after translation and validation.

Inclusion criteria were patients with moderate or severe LUTS (International Prostate Symptom Score [IPSS] >16), failure of previous medical treatment with a washout period of at least 2 wk, maximum flow rate (Qmax) <15 ml/s, and ability to give a fully informed consent. Exclusion criteria were patients with urethral strictures, bladder stone, or neurogenic bladder. In case of an elevated PSA level or abnormal digital rectal examination, at least two series of TRUS-guided prostate biopsies were performed preoperatively to rule out prostate cancer (PCa). Patients diagnosed with Pca were excluded from the study. Patients with known neurological disorders such as multiple sclerosis, Parkinson’s disease, or a known history of spinal cord injury were also excluded. The use of anticoagulants or indwelling urinary catheters for urinary retention was not a criterion for exclusion.

The patient ages were 70 to 96 years old and the average age was 82.8 ± 8.6 years old. There were 1–8 internal diseases as the concomitant illnesses and there were 88 cases with abnormal ECGs for the complications. Of these, there were 42 cases of cardiovascular disease, 56 cases of hypertension, 51 cases of respiratory system diseases, 39 cases of cerebrovascular diseases, 24 cases of anemia, 16 cases of liver or kidney dysfunction, 18 cases of diabetes and 15 cases of hypoproteinemia. The average number of complications was 3.7 internal diseases. There were 8 cases of patients with simultaneous bladder stones, and some of the patients had as complications fever, hypovolemia, acid–base imbalance and hyperlipidemia, etc. A comprehensive and systematic assessment was conducted for all patients, including a digital rectal examination, urinalysis, transrectal ultrasound for measuring the prostate size, urinary system examination, serum PSA examination, etc., and IPSS scoring was conducted prior to surgery in each case. A prostate puncture biopsy was conducted for patients with an abnormal PSA level or digital rectal examination abnormalities in order to eliminate prostate cancer. The average preoperative prostate volume was 66.1 ± 25.3 (30–160) ml, and the residual urine volume (RUV) was (246 ± 38) ml. There were 20 cases for which bladder fistulization and an indwelling catheter were performed before the surgery, and the maximum urine flow rate (Qmax) was 3.6-10.4 (6.2 ± 2.6) ml/s. The average International Prostate Symptom Score (IPSS) was (26.5 ± 3.8) points. Prior to the operation, antihypertensive and antidiabetic drugs were applied to lower the blood pressure to 150/95 mmHg and under, and the blood sugar to 8.4 mmol/L and under. After those patients with kidney failure were treated with an indwelling catheter, the serum creatinine level was at 330 μmol/L and under, and an assessment of the anesthesia risk was conducted by the anesthesia department and related departments. This study was conducted in accordance with the declaration of Helsinki. This study was conducted with approval from the Ethics Committee of 307 Hospital of PLA Written informed consent was obtained from all participants.

### Anesthesia method

Total intravenous anesthesia with a Proseal laryngeal mask airway (Ningbo Boya Medical Equipment Co., Ltd) was adopted, and the patients assumed the lithotomy position. A routine multiparameter monitor monitored the ECG, heart rate, respiration, oxygen saturation and urine volume, and at the same monitoring of such indices as the invasive artery blood pressure, central venous pressure, etc. was carried out. The chief medications for induction and maintenance were Propofol, Fentanyl, Midazolam and Vecuronium (or Atracurium).

### Surgical methods

The non-contact green laser treatment system made by Green Light Corporation (USA) was employed. The power was 120 watts. The side firing ADD Stat™ laser fiber with a 600 mm core diameter was placed in the cavity with the double sheath mirror (22.5 Fr, 30°) (Karl Storz Karl Storz GmbH & Co. KG), and physiological saline was the irrigation fluid. The operation was conducted under monitoring with a Storz camera system, the ADD non-contact optical fiber issued red light as the pilot light, and the distance between the optical fiber side hole and the prostate tissue was approximately 0.5 mm (whenever hemorrhaging occurred, it can be enlarged to 2–3 mm or the laser output power can be adjusted to 40 watts). The vaporization begins from the median lobe at the bladder neck, the ADD optical fiber is continuously swept left and right, vaporization gradually proceeds up to the verumontanum, place the bottom part of the anterior urethra in the same plane as the bladder trigone, and separately vaporize the lobes on both sides as much as possible up to the prostate capsule. Among the patients, there were 8 cases with complications of bladder stones, for which first the PVP surgery was conducted and then pneumatic lithotripsy (EMS) was conducted, and an Eric flushing device [Hangzhou Tonglu medical optical instrument factory] sucked out the bladder stone remnants. After the operation a three cavity balloon urinary catheter was left to indwell, and a decision was made about whether or not to perform bladder irrigation based on observations of the patient’s urine drainage.

### Observation index

The parameters that we assessed chiefly included the surgical operating time, amount of energy consumed, changes in the postoperative hemoglobin and serum sodium, was there urethral catheterization or not, hospitalization time and early complications during and after the operation. The patient follow up visit times were 1, 3, 6 and 12 months after the operation, and then once annually thereafter. In addition to recording the occurrence of adverse events, the follow up visit contents also included the maximum urine flow rate (Qmax), residual urine volume (RUV) and postoperative PSA changes. During the course of follow-up, filling in the PISS [sic] score sheets for all of the patients was required. In addition, we arranged the prostates with differing sizes in groups (smaller than 80 ml, larger than 80 ml), and thereby assessed the surgical effects based on prostate size and the symptom alleviation time.

### Statistic analysis

All of the data were indicated as the average number + standard deviation (SD), SPSS 15.0 was used for the statistical analysis, and a comparative analysis between three groups or even more groups was done with a Kruskal-Wallis-H test. A Wilcoxon test was done for the (IPSS, Qmax, Vres and PSA) statistical analyses within the different groups. A chi-square test was employed for the comparative analysis of complications. *P* < 0.05 was regarded as statistical significance.

## Results

### Basic conditions of patients

From January 2009 to January 2011, PVP surgery was conducted on 120 patients with a 120 watt green laser. Table 1 shows the basic patient data. Of all the patients, 30% took an oral anti-coagulant drug (aspirin, Clopidogrel, coumarin derivatives). There were 88 cases with abnormal ECGs for the complications and 1–8 internal diseases as the concomitant illnesses, and the average number of complications was 3.7 internal diseases. Of these, there were 42 cases of cardiovascular disease, 56 cases of hypertension, 51 cases of respiratory system diseases, 39 cases of cerebrovascular diseases, 24 cases of anemia, 16 cases of liver or kidney dysfunction, 18 cases of diabetes and 15 cases of hypoproteinemia. There were 8 cases of patients with simultaneous bladder stones, and some of the patients had as complications fever, hypovolemia, acid–base imbalance and hyperlipidemia, etc. There were no significant differences between the ages and PSA levels among the patients with differing prostate sizes in the two groups.

**Table 1:** Analysis of perioperative parameters before PVP surgery was performed on 120 Patients

	**Number of patients**	**Age (year)**	**IPSS**	**Qmax (ml/s)**	**PVR (ml)**	**Prostate volume (ml)**
Prostate Volume	40	78.6	25.6	7.6	249.5	25.1
Smaller than 80 ml						
Prostate Volume	80	84.9	26.95	5.5	362.2	86.6
Larger than 80 ml						
Not anti-coagulant	84	81.1	26.4	7.5	256.2	66.5
Anti-coagulant	36	86.6	26.7	3.1	260.7	35.1
No Indwelling Catheter	100	82.1	25.9	7.44	156.5	61.6
Indwelling Catheter	20	86.06	29.5	NR	719.0	88.6

### Perioperative data

The amount of energy consumed during the operation and the prostate volume were clearly correlated (*P* < 0.001), the average indwelling catheter time for all patients was 1.67 ± 1.2 days, and the average hospitalization time was 4.67 ± 2.9 days.

### Recovery of functions

The average follow-up time was 20.8 ± 3.2 (18–24) months. After the urinary catheter was removed, the urination symptoms of the majority of patients was remarkably improved compared to those before the surgery, and during the follow-up period of up to 2 years there was a marked improvement in all the patients’ subjective and objective test parameters. There was no marked distinction in the maximum urinary flow rate test for each group of patients with different prostate sizes (Table 2).

**Table 2:** Analysis of perioperative parameters for prostates VP surgery with differing volume size

**Basic parameters**	**Prostate volume**		**Total average number**	***P *** **value**
	**Smaller than 80 ml**	**Larger than 80 ml**		
Surgery time	61.59 ± 19.2 (20–100)	86.0 ± 36.4 (50–150)	76.4 ± 25.7 (20–150)	<0.001
Amount of energy consumed	111 ± 77 (31–550)	265 ± 85 (120–596)	224 ± 85 (31–596)	<0.001
Indwelling catheter time	1.6 ± 0.8 (1–4)	1.7 ± 1.0 (1–5)	1.67 ± 1.2 (1–5)	Ns
Hospitalization time	2.8 ± 1.8 (1–6)	3.5 ± 2.2 (1–10)	4.67 ± 2.9 (1–10)	<0.01
Hemoglobin test (g/d)				
Preoperative	12.6 ± 2.1	11.8 ± 3.5	12.06 ± 2.1	Ns
Postoperative	12.1 ± 3.4	11.2 ± 3.2	11.5 ± 1.2	Ns
Serum creatinine (mmol/L)				
Preoperative	124 ± 3.8	126 ± 3.6	125 ± 2.8	Ns
Postoperative	123 ± 3.1	121 ± 3.4	122 ± 3.2	Ns

### Analysis of complications

The surgical complications may be classified as three kinds, the complications during surgery, early postoperative complications (within 12 weeks after surgery) and those during the follow-up period. Table 3 shows a summary of the complications during the 24-month follow-up period after surgery for prostates of differing sizes.

**Table 3:** Analysis of perioperative complications

**Time point**	**Complication**	**Patient’s preoperative condition**					
		**No indwelling catheter N (%)**	**Indwelling catheter N (%)**	**No anti-coagulation treatment N (%)**	**Anti-coagulation treatment N (%)**	**Prostate volume (< 80 ml) N (%)**	**Prostate volume (≥ 80 ml) N (%)**
	Required electric coagulation	0	0	0	0	0	0
Perioperative period	Capsule perforation	_1 (1)_	0	_1 (1.1)_	0	_1 (2.5)_	0
	Blood transfusion	0	0	0	0	0	0
	TURP syndrome	0	0	0	0	0	0
	Acute renal failure	_0_	_1 (5)_	_0_	_1 (2.7)_	0	_1 (1.25)_
	Bladder irrigation	_5 (5)_	_9 (45)_	_6 (7.1)_	_8 (22.2)_	_4 (10)_	_10 (12.5)_
	Early dysuria (3–14 days)	_11 (11)_	_7 (35)_	_13 (15.5)_	_5 (13.9)_	_8 (20)_	_10 (12.5)_
	Re-catheterization	_1 (1)_	_1 (5)_	_1 (1.2)_	_1 (2.7)_	_1 (2.5)_	_1 (1.25)_
	Urinary tract infection	_3 (3)_	_2 (10)_	_3 (3.6)_	_2 (5.5)_	_2 (5)_	_3 (3.75)_
	Severe dysuria	_1 (1)_	_1 (5)_	_2 (2.4)_	0	_1 (2.5)_	_1 (1.25)_
Early postoperative (12 weeks)	Blood transfusion	0	0	0	0	0	0
	Interruption of urination	_1 (1)_	_1 (5)_	_1 (1.2)_	_1 (2.7)_	_2 (5)_	0
	Reoperation	_2 (2)_	_2 (10)_	_3 (3.6)_	_1 (2.7)_	_0_	_3 (3.75)_
	Urethral stricture	_0_	0	_0_	0	_0_	0
	Bladder neck contracture	_0_	_0_	_0_	_0_	_0_	_0_
	Urinary incontinence	_2 (2)_	_1 (5)_	_1 (1.2)_	_3 (8.3)_	_2 (5)_	_2 (2.5)_
Follow up (6 months)	Interruption of urination	_1 (1)_	_1 (5)_	_1 (1.2)_	_1 (2.7)_	_2 (5)_	0
	Reoperation	_2 (2)_	_2 (10)_	_3 (3.6)_	_1 (2.7)_	_1 (2.5)_	_3 (3.75)_
	Urethral stricture	_1 (1)_	0	_1 (1.2)_	0	_1 (2.5)_	0
	Bladder neck contracture	_1 (1)_	_1 (5)_	_1 (1.2)_	_1 (2.7)_	_1 (2.5)_	_1 (1.25)_
	Urinary incontinence	_2 (2)_	_1 (5)_	_1 (1.2)_	_3 (8.3)_	_2 (5)_	_2 (2.5)_
Follow up (12–24 months)	Interruption of urination	_1 (1)_	_1 (5)_	_1 (1.2)_	_1 (2.7)_	_2 (5)_	0
	Bladder neck contracture	_2 (2)_	_2 (10)_	_3 (3.6)_	_1 (2.7)_	_1 (2.5)_	_3 (3.75)_
	Urinary incontinence	_1 (1)_	0	_1 (1.2)_	0	_1 (2.5)_	0
	Bladder neck contracture	_1 (1)_	_1 (5)_	_1 (1.2)_	_1 (2.7)_	_1 (2.5)_	_1 (1.25)_
	Urinary incontinence	_2 (2)_	_1 (5)_	_1 (1.2)_	_3 (8.3)_	_2 (5)_	_2 (2.5)_

### Analysis of complications during surgery

No serious complications during surgery manifested themselves during the surgical period. None of the patients manifested hyponatremia or any expression of excess fluid absorption during surgery. No major hemorrhaging or recurrent bleeding was observed either during the surgery or postoperatively, and no instances of blood transfusion occurred. The bleeding was relatively great in 12 patients (10%) during the course of the surgery, and unclear vision manifested itself during the course of the surgery and the operation time was prolonged, but none of the patients suffered any serious complications postoperatively. In 12 patients (10%) it was necessary to carry out vaporization with 2 or more optical fibers since the optical fiber was damaged and the prostate volume was too large.

### Early postoperative complications

Mild hematuria manifested itself in 35 patients (29.2%) postoperatively, so bladder irrigation was conducted. Of these, there were 68.5% who were currently taking an oral anti-coagulant drug. Acute renal failure occurred in 1 case (0.83%) on Day 1 after the operation, and it was necessary to carry out hemodialysis, and the patient’s renal function completely recovered within 1 month after the operation. The incidence of postoperative dysuria was approximately 15% (18 cases).

### Follow up

During the 24-month follow up period, we observed 4 patients (3.3%) for whom we needed to carry out vaporization treatment another time. The incidence of urethral stricture was 0.8% (1/120), and the incidence of bladder neck contracture was approximately 1.7% (2/120), where in the incidence in patients whose prostate volume was relatively small was higher (*P* < 0.05). The incidence of stress urinary incontinence was approximately 2.5% (3/120), and the patients’ ages were all greater than 80 years old.

## Discussion

TURP was the gold standard of BPH operation, it’s efficiency and safety had been confirmed clinically. Cleynenbreugel et al. [9] compared four prospective studies with PVP with TURP. The outcome parameters of the two methods were no significant difference [10]. But, compared with PVP, patients undergoing TURP experienced more severe adverse effects. Al-Ansari [11] first used RCT comparing 120-W PVP versus TURP with a long-term follow-up of 36 mo. they found that 20% of patients in the TURP group developed significant bleeding, confirming that bleeding is a major complication after TURP and often requiring transfusion.

Patients who are on oral anticoagulant therapy have a high complication rate with a transfusion rate of 30% when performing conventional TURP [12]. But the PVP laser causes very little bleeding, so it can be used successfully in patients on anticoagulant therapy [13,14]. In my study, although 30% of the patients took an oral anti-coagulant drug, no major hemorrhaging or recurrent bleeding was observed either during the surgery or postoperatively, and no instances of blood transfusion occurred. Patients in the TURP surgery will experience incidence of 5% TUR syndrome [15]. In contrast, compared with the preoperative levels, none of my clinic’s patients in the PVP operation developed significant change in the hemoglobin or sodium levels. This could be explained by the fact that PVP uses saline as an irrigant, thus almost removing the risk of TUR syndrome.

We employed total intravenous anesthesia with a Proseal laryngeal mask airway (PLMA) for the above-described elderly patients, and were able to obtain good anesthetic effects through the method of a drug combination. The Proseal laryngeal mask airway has the advantages that it is easy operate, it does not cause mechanical damage to the vocal cords or trachea, the stress response is light, and it is possible to maintain ventilation under conditions of voluntary respiration. It is beneficial for elderly patients with preoperative complications of hypertension, cardio- and cerebrovascular diseases and respiratory failure, and its unique double balloon structure and drain tube structure raise the hermetical seal quality of the laryngeal mask airway, and can also extract gastric fluid or gas, and can reduce complications due to gastric distension and reflux and mis-aspiration of gastric contents. It also lowered complications such as cardio- and cerebrovascular accidents caused by abrupt fluctuations in circulation and postoperative pulmonary infections [16].

We employed the following methods to handle the frequently observed complications in PVP surgery.

### Hemorrhage

Even the PVP laser causes very little bleeding during the operation. In my clinic’s patients, the bleeding was relatively great in 12 patients (10%) during the course of the surgery. Hemorrhage during green laser surgery can be classified in two types, venous hemorrhage and arterial hemorrhage. Venous hemorrhage chiefly originates in the squeezing of the urethral mucous membrane and hemorrhage inside the prostate tissue. The volume of such hemorrhaging is not very large, but it will have an effect on the field of view during the course of the operation, so during the course of insertion of the mirror sheath insertion is done along the urethra to the maximum extent possible, and during the course of the operation the optical fiber is moved as much as possible, in order to reduce the activity of the mirror sheath from being able to reduce the generation of bleeding. Attention is particularly needed at the time of bladder neck incision, especially since the prostate tissue at this place hemorrhages easily, so we recommend that the speed during vaporization be lowered so that it is possible to reduce hemorrhaging.

Rupture of the artery supplying blood to the prostate tumor is the chief cause of arterial hemorrhage, and avoiding the prostate stones during the course of the operation can reduce the occurrence of this kind of hemorrhage [16]. If one wishes to control hemorrhage, the best method is to use 20 watts condensing power to illuminate the tissue in the area around the hemorrhage locus, and to make the tissue edema place pressure on the artery and achieve the goal of hemostasis.

### Dysuria

The incidence of postoperative dysuria was approximately 15% (18 cases) in my clinic’s patients. Comparatively speaking, patients whose prostate volume was smaller have higher proportion of dysuria (*P* < 0.01), and ordinarily it was not until 4–6 moths after the surgery that the symptoms of dysuria could be resolved completely. The chief causes of postoperative dysuria are a decline in laser vaporization efficiency, incomplete tissue vaporization and an excessively high coagulative prostate proportion, and these are positive correlated with the extent of a patient’s dysuria. Therefore, during the course of the operation one should avoid as much as possible excessive cauterization of the bladder trigone. Since patients with excessively large prostate medial lobes have to undergo repeated cauterization and hemostasis during the operation, this results in chronic inflammation, fibrosis and calcification of the remaining prostate tissue, and this raises the risk that dysuria will occur postoperatively, and one should consider using Dexamethasone to alleviate the symptoms in Week 1 after the operation.

### Bladder neck contracture

The incidence of bladder neck contracture was approximately 1.7% (2/120) in my clinic, where the incidence was higher in patient with smaller prostate (*P* < 0.05). When we conducted a comparison of prostates with different volumes, we were able to learn that the incidence of bladder neck contracture was markedly lower in the group whose prostate volume was relatively large. Excessive cauterization of the bladder neck may cause bladder neck contracture [17]. Therefore, during the course of the operation one should avoid as much as possible excessive coagulation and laser cauterization. Using the optical fiber as much as possible at the bladder neck to replace the entry and exit activity of the mirror sheath can reduce the damage to the bladder neck. It is necessary to carry out preventively a bladder neck incision during the course of PVP surgery in order to prevent contracture from manifesting itself postoperatively.

### Urethral stricture

Urethral stricture may cause by the injury of the bulbous part of the urethra. We carried out the operation with a 22.5 F laser mirror equipped with a single optical fiber channel, and the incidence of urethral stricture within 1 year after the surgery was only 0.8% (1/120), and there was a marked decline in the urethral stricture rate compared to previous literature reports. During the course of inserting the laser mirror sheath, one should try to the extent possible to achieve entry under direct vision, and avoid damaging the urethral mucous membrane. It is also extremely important to carry out preventive handling if one discovers urethral stricture prior to the operation. By changing the 26 F mirror sheath that was initially used to a 22.5 F laser vaporization sheath, it is possible to markedly reduce the incidence of urethral stricture. During a randomized comparative study the 22.5 laser sheath and TURP, one can see that there is a higher probability of urethral stricture occurring in the TURP operation (6.1% vs. 2.1%).

The reason why the incidence of urethral stricture is relatively high in patients whose prostate volume is relatively small (< 40 ml) may be that the initial researchers using PVP usually started from small prostates, and in order to keep the urinary sphincter from suffering any damage, when they moved the laser mirror sheath it was even closer to the bulbous part of the urethra, and this may have caused damage to the mucous membrane, and ultimately caused urethral stricture.

### Damage to the ureteral opening

There wre only 1 case of patient need hemodialysis after PVP operation because of damaging the ureteral opening. The chief cause of damage to the ureteral opening is that the laser beam has irradiated ureteral opening during the operation. Therefore, it is extremely important to check the location of the ureteral opening before the start of laser vaporization, and the ureteral opening must be examined again prior to handling the bladder neck and prostate medial lobes. It is possible to label the ureteral opening beforehand with indigo carmine, and if the prostate is too large and is placing pressure on the bladder neck, [the laser beam] suddenly enters the bladder and covers the ureteral opening at both sides, and damage to the ureteral opening will appear easily. Therefore, it is necessary to set the laser beam position first before the prostate medial lobes and lateral lobes prior to vaporization.

### Reoperation for patients with large volume prostate hyperplasia

In my study, there were 4 patients (3.3%) needed to carry out vaporization treatment another time. The chief reasons were that the prostate tissue that was vaporized in the first operation was insufficient and the prostate tissue grew again. The preoperative prostate volume of patients who underwent a second operation was relatively large in each case.

Having adequate laser power acting on the prostate is the key to the therapeutic effects of PVP surgery. During the course of our study, the reoperation rate was approximately 4% or less, and among our patients the reoperation rate for patients with larger prostate volume was higher.

The results of study indicate that the total energy consumption of patients with a relatively large prostate was relatively high, whereas the average energy per each millimeter of prostate was relatively low. In addition, the surgeon’s learning curve may also affect the reoperation rate. In the course of reviewing the study, one can see that the reoperation for the first 30 patients was 10%, while the reoperation rate for the patients after that rapidly declined to 0-1%. When it is a prostate with a large volume, it may be necessary to consume two or more optical fibers before it is possible to obtain a suitable urination cavity path. The costs of the surgery increase for these patients, but PVP surgery reduced each of the complications of such patients compared with an open operation, and it is also worth considering such problems as the prolongation of the hospitalization time, loss of the ability to work and needing to convalesce for a relatively long time.

## Conclusion

PVP surgery was safe and effective. There is little hemorrhaging preoperatively and postoperatively, and there were no cases of blood transfusion. The operation time was controlled to 150 minutes or less, and it was possible to improve the various subjective and objective urination parameters. When it comes to various high risk patients and patients taking an oral anti-coagulation drug, 120 watt PVP surgery can minimize hemorrhaging and various surgery-related risks, reduce complications like urinary incontinence and urethral stricture, and improve markedly the postoperative urination symptoms. The results of this study indicate clearly that therapeutic effects of 120 watt PVP surgery are excellent and the effects of treatment are good, and are more suitable for use in high risk patients. Complications are not classified using a standardized method is the limition of my study.

## Competing interest

The authors declare that they have no competing interest.

## Authors’ contributions

Dr Mai and Dr Chen carried out this clinic studies, participated in the operation and drafted the manuscript. All authors have read and approved the final manuscript.

## Authors’ information

Lijun Chen and Haixing Mai are co-first authors.

## Pre-publication history

The pre-publication history for this paper can be accessed here:

http://www.biomedcentral.com/1471-2490/13/64/prepub
